# Gradient Internal Standard Method for Absolute Quantification of Microbial Amplicon Sequencing Data

**DOI:** 10.1128/mSystems.00964-20

**Published:** 2021-01-12

**Authors:** Shilei Wang, Qun Wu, Ying Han, Rubing Du, Xiaoyong Wang, Yao Nie, Xiaowei Du, Yan Xu

**Affiliations:** a State Key Laboratory of Food Science and Technology, Synergetic Innovation Center of Food Safety and Nutrition, School of Biotechnology, Jiangnan University, Wuxi, Jiangsu, China; b Technical Center, Xinghuacun Fenjiu Distillery Co. Ltd., Fenyang, Shanxi, China; Purdue University

**Keywords:** internal standards, absolute quantification, microbiota, solid-state fermentation

## Abstract

High-throughput amplicon sequencing is a critical tool for studying microbiota; however, it results only in relative abundance data. Thus, changes in absolute abundance of microbiota cannot be determined, which hinders further microbiology research. We have therefore established a gradient internal standard absolute quantification (GIS-AQ) method to overcome this issue, which can simultaneously obtain the absolute abundances of bacteria and fungi. Deviations from the quantitative equations of microbes and internal standards were eliminated through calibration. Compared with traditional quantitative real-time PCR and microscopy quantifications, this method is reliable (*R*^2^_average_ = 0.998; *P < *0.001) and accurate (*P*_internals versus microscopy_ > 0.05). The GIS-AQ method can be adapted to any amplicon primer choice (e.g., 336F/806R and ITS3/ITS4), rendering it applicable to ecosystem studies including food, soil, and water samples. Crucially, when using solid-state fermentation samples from various temporal dimensions, the results obtained from the relative and absolute abundance are different. The absolute abundance can be used to study the difference in communities between different samples, and the GIS-AQ method allows this to be done rapidly. Therefore, combining the absolute abundance with relative abundance can accurately reflect the microbiota composition.

**IMPORTANCE** To solve the problem of amplicon sequencing cannot discern the microbiota absolute abundance, we proposed a gradient internal standard absolute quantification method. We used Chinese liquor fermentation as a model system to demonstrate the reliability and accuracy of the method. By comparing the relative and absolute abundances of microbiota in various temporal dimensions, we found dynamic changes in the absolute abundance of communities under various temporal dimensions from the relative abundance. Based on its design principle, this method can be widely applied to different ecosystems. Therefore, we believe that the GIS-AQ method can play an immeasurably useful role in microbiological research.

## Abstract (precis)

To solve the problem of amplicon sequencing cannot discern the microbiota absolute abundance, we proposed a gradient internal standard absolute quantification method. We used Chinese liquor fermentation as a model system to demonstrate the reliability and accuracy of the method.

## INTRODUCTION

The study of microbiota such as those in soil (1), diseases (2), fermented food (3), and sites of human activity (4, 5) has paid greater attention to various temporal dimensions, such as seasonal (6) and age (7) differences. Comparing microbial abundances between different samples in various temporal dimensions is important for expanding the breadth and depth of the research.

Omics techniques have been utilized as tools in microbiology (8, 9). Amplicon sequencing is the most common method for analyzing microbial community structures (10); however, this method can generate spurious results (11). The relative abundance does not reflect the variation in absolute abundances of the individual microbes (12). Thus, to compare samples across temporal and spatial dimensions, we must use microbiota absolute quantitative results (13).

The internal standard can be utilized to solve problems with absolute microbial quantification (12). Recently, researchers have conducted quantitative analyses of microbiota abundances through the synthetic internal standards of 16S rRNA, 18S rRNA, and internal transcribed spacer (ITS) (14) using a standard external strain, such as a fluorescently labeled strain (15). Presently, only one internal standard can be added to ecosystems with only one concentration. However, the different individual microbes in the microbiota cover a wide range of concentrations in the actual samples (16, 17). For example, the concentrations of different bacteria ranged from 6.9 × 10^4^ CFU g^−1^ to 6.5 × 10^8^ CFU g^−1^ in compost (18). Typically, the closer the internal standard is to the microbial concentration, the more accurate the quantitative result is. Studies have indicated that different results are obtained using internal standards with various concentrations, and only 20 to 80% of microbes can be accurately quantified (14). Hence, to quantify different microbial absolute abundances, it was suggested to determine the same sample multiple times by adding different internal standard concentrations at various times, which is time-consuming. As a result, it remains challenging to accurately determine microbes with different concentrations by measuring a sample only once. Therefore, it is important to establish an efficient method to accurately quantify microbes with large concentration ranges in complex microbiota.

In this study, we established a gradient internal standard absolute quantification (GIS-AQ) method to absolutely quantify microbes in complex microbiota in solid-state fermentation. The gradient internal standard group concentrations were simultaneously added to the same sample to quantify microbial concentrations of different orders of magnitude. The specific primers of the 16S rRNA, ITS, and internal standards were designed on the same sequence using one sequence fragment to reduce the number of internal standards added. Moreover, the deviations from the quantitative equations of microbes and internal standards were eliminated through calibration. In addition, we built a mock community with specific primers for each organism in the communities with quantitative real-time PCR (qPCR) validation. Based on the mock community, we demonstrated the reliability and accuracy of the GIS-AQ method by microscopy quantification (the microbial gradient dilution solution was counted using optical microscopy). Finally, the GIS-AQ method was used to identify the absolute abundance of microbiota during solid-state Chinese liquor fermentation. This method is useful for quickly understanding the absolute abundance of microbial communities over different temporal dimensions and can play an immeasurable role in microbiological research.

## RESULTS

### GIS-AQ method principle.

**FIG 1:**
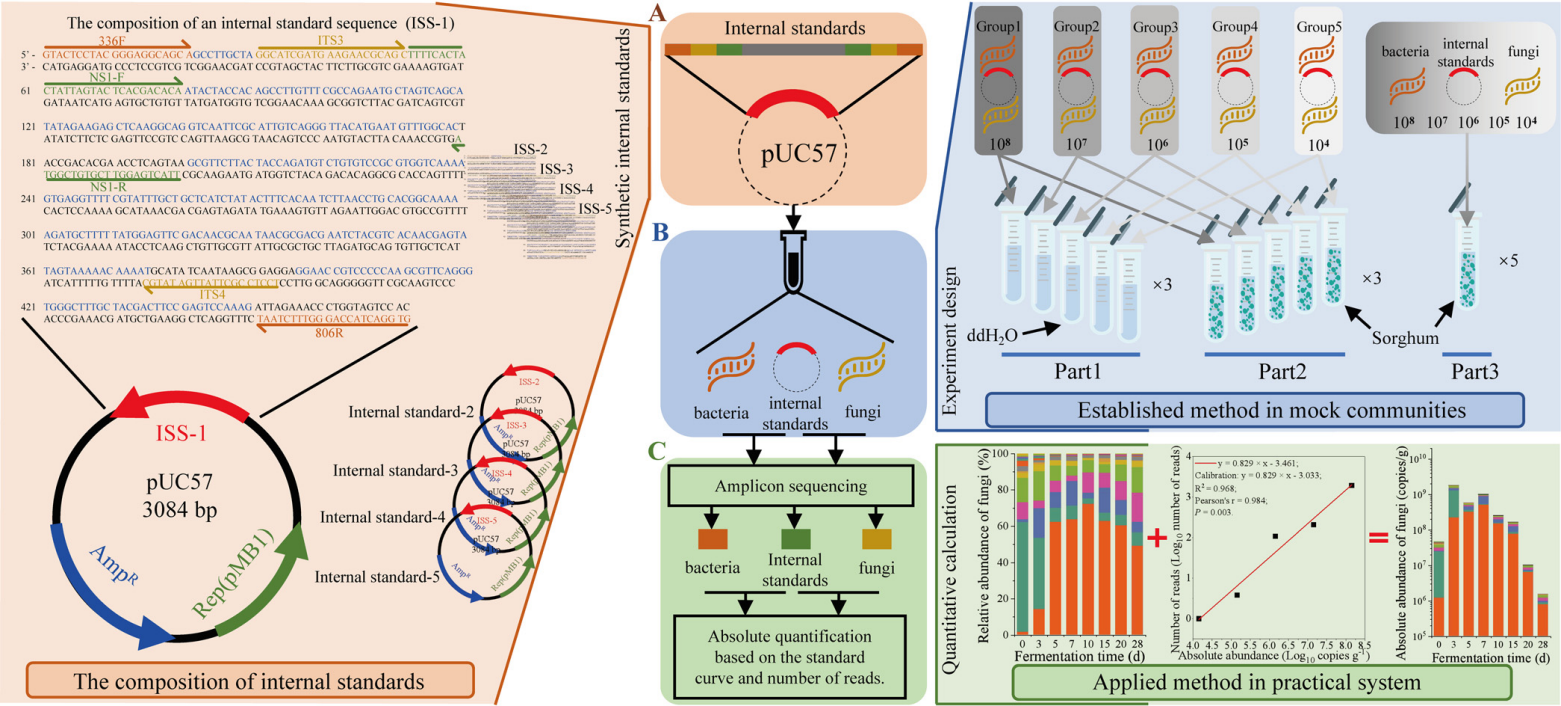
Design and application method of internal standards. (A) Synthetic internal standards. The internal standards were designed and constructed to contain different primer sequences (336F/806R, ITS3/ITS4, and IS-F/IS-R) and a specially designed gene fragment. (B) Experiment design. Three experiments were designed to demonstrate the accuracy of the internal standards. (Part 1) The internal standards and microbes were added to the ddH_2_O. (Part 2) The internal standards and microbes were added to the sorghum. (Part 3) Five internal standards and 10 microbes were added to the sorghum. (C) Quantitative calculation. Based on the traditional amplicon sequencing results and the quantitative equation obtained in part 3, the microbial absolute abundance in the sample was calculated.

First, we added five different internal standards (pUC-57 plasmid) with a 10× concentration gradient to a sample to construct a gradient internal standard group for absolute quantification (Fig. 1A). Each internal standard contained a specific pair of primers and recognition sequences (Table 1) that were not present in the microbial genomes based on a search of the National Center for Biotechnology Information (NCBI) database and were flanked by the universal primers of the bacterial 16S rRNA V3-V4 and fungal ITS2 regions (Fig. 1A). The concentration of plasmid was calculated by the calculation equation in Materials and Methods. Second, before genomic DNA extraction, five internal standards (plasmid [Fig. 1A]) were added to the same sample at different concentrations to construct the concentration gradient (approximately 10^4^, 10^5^, 10^6^, 10^7^, and 10^8^ copies g^−1^). The concentrations were chosen based on previous studies on the microbial concentration ranges (10^3^ to 10^9^ copies g^−1^) in the Chinese liquor solid-state fermentation ecosystem (19–21) (Fig. 1B). Due to the specific primers and recognition sequences (see Materials and Methods) of the five internal standards, the internal standard sequences (ISS) were easy to identify during data analysis. Third, there was a positive correlation (Pearson’s *r* > 0.9; *P < *0.05) between the copies of the internal standards added and detected (Fig. 1C). The numbers of reads (log_10_ reads) were normalized to the concentration (log_10_ copies per gram) based on their linear correlation (raw data converted by log_10_) to complete the individual microbial quantification.

**TABLE 1:** Internal standard sequences^*a*^

Internal standard	Sequence length (bp)	% GC	Complete internal standard sequence
IS1	472	46.40	5′-GTACTCCTACGGGAGGCAGCAGCCTTGCTAGGCATCGATGAAGAACGCAGCTTTTCACTACTATTAGTACTCACGACACAATACTACCACAGCCTTGTTTCGCCAGAATGCTAGTCAGCATATAGAAGAGCTCAAGGCAGGTCAATTCGCATTGTCAGGGTTACATGAATGTTTGGCACTACCGACACGAACCTCAGTAAGCGTTCTTACTACCAGATGTCTGTGTCCGCGTGGTCAAAAGTGAGGTTTTCGTATTTGCTGCTCATCTATACTTTCACAATCTTAACCTGCACGGCAAAAAGATGCTTTTTATGGAGTTCGACAACGCAATAACGCGACGAATCTACGTCACAACGAGTATAGTAAAAACAAAATGCATATCAATAAGCGGAGGAGGAACCGTCCCCCAAGCGTTCAGGGTGGGCTTTGCTACGACTTCCGAGTCCAAAGATTAGAAACCCTGGTAGTCCAC-3′
IS2	472	46.20	5′-GTACTCCTACGGGAGGCAGCAACTCCCTGTTGCATCGATGAAGAACGCAGCAAGAATGATGATAATCATGAGTACTGTGCTAAGACGGTGTCGAAACAAAGCGGTCTTACGGTCAGTCGTATTTCCTCTCGAGTCTCGTCCAGTTGAGCGTATCACTCTCAATGTACTAGCAAGCCAAGAAGGCTGTGCTTGGAGTCAATCTGATGTAGGATGATCTCCAGACACCAGGCCACTACTCTTCATACTTAAAGCATAAACGTCGAACAGTCATGAAAGTCTTAGTACCGGACGTACCATTTTACTGTGAATATTACCTGAAGCTGTACCGTTATTGAGGAGCAAAGATGTAGTACTGCTCTTATCATATTTGTATTGGCATATCAATAAGCGGAGGACCCACGCACCTGATCGCTCCTCGTTTGCTTTTAAGGACCGGACGAACCACAGAGCATTAGAAACCCTGGTAGTCCAC-3′
IS3	472	45.60	5′-GTACTCCTACGGGAGGCAGCATACTGCGACCGCATCGATGAAGAACGCAGCCTCTAACTACTATCAATACCCATGACTTGACTCTGCTGCAGCTACGTATCGCCTGAAAACCAGTTAGTGTTAAGGAATGCTCTGACCAGGACAACACACGTAGTGAAAGTTACATGTTCGTTGGGTTCTTCCGACTCGGATCTGAGTTGACCAATGACTCACTTGAGATCTGAACCCTAGTGATGATAAATATGTATCTCGTTCACGCAGATTGCCAGCACTTTCAGAATCATGATGTGCATGGTAGAATGACTCTTATAACGAACTTCGACATGATAATATCCCCCCCTTTCAACTTCTAGAGAAGAAAAGTATTGACATGAGGCATATCAATAAGCGGAGGATTCATCAGCTAACGTAACGGTTAGAGGCTCGCTAAATCGCACTGTCGGCGTCCCTATTAGAAACCCTGGTAGTCCAC-3′
IS4	472	45.40	5′-GTACTCCTACGGGAGGCAGCATGGGTATTTTGCATCGATGAAGAACGCAGCCGTTCCCAGCACAACAGCCAAAGAAGTTTCCAATTTTTTATTTCCGAATGACATGTGTCTCCTTGCGGGTAAATCGCCGACCGCAAAACTTAGGAGCCAGGGGAAACAGATAGGTCTAATTAACTTAAGGGAGTAAATCTTGGAATCGTTCAGTTGTAACTATATACTTACGCTGGAACTTCTCCGGCGAATTTTTACTGTCACCAACTACGAGATTTGAAGTAAACCAATTAAGCACATAGTCGCGCTATCCGACAATTTCCAAATTATAACATATCGTTCCATGAAGGCCAGAATTACTTACCGGCCCTTTCCATGCGTGCAGCATATCAATAAGCGGAGGAGCTGATCCGAGTCGAGTTAAAAACACCAGTACCCAAAACCAGGCGGGCTCGCCACATTAGAAACCCTGGTAGTCCAC-3′
IS5	472	46.90	5′-GTACTCCTACGGGAGGCAGCAGTCGGCTAATGCATCGATGAAGAACGCAGCCCATACCCTCCTACTTCCCCGCTTATCTATCCGAAGAGAGAGTGTGCGATCCTCCGTTAAGATATTCTTACGTATGATATAGCTATGTATTTTGTAGAGGTAGCGAACGCGTTAAACATTTCACAGATAGTGGGGATTCGGGCAAAGGGCGTATAATTGTGGACTAACATAGTCGTAAACTACGATGGTACCAACTCAATCTCAGCTCGTGCGCCTAAATAACGTACTCATCTCAACTGATTCTTGGCAATCTACGGAGCGACTTGATTATTAACAGTTGTCTAGCGAGTTCTAATCTTTTACCAACATCGTAATAGCCTCCAAGCATATCAATAAGCGGAGGAATCCGCAGTGGCCGGTAGACACACGTCCACCCCGCTGCTCTGTGACGGGGACTAAATTAGAAACCCTGGTAGTCCAC-3′

a The bold sequences are the primers 336F/806R. The italicized sequences are the primers ITS3/ITS4. The underlined sequences are the specific internal standard sequence primers.

### Detection of the microbial absolute abundance in mock communities using the GIS-AQ method.

To explore the possibility of quantifying bacterial and fungal communities using internal standards, we used five bacteria and five fungi to build a mock community (see Materials and Methods). Using the mock community, we established a gradient internal standard absolute quantification (GIS-AQ) method and demonstrated its accuracy using the following five steps.

First, we tested whether the five internal standards were stable and reliable (Fig. 1B, part 1; see Materials and Methods). Based on the qPCR results, the five parallel experiments demonstrate that there were no significant differences (*P > *0.05) and the quantitative equation was stable and reliable (*R*^2^ = 0.999; *P < *0.001; see Fig. S1 in the supplemental material) for use in this method.

10.1128/mSystems.00964-20.1 FIG S1 Reliability test of five internal standards. The red band marks the 95% prediction of the linear equation. Download FIG S1, PDF file, 0.01 MB. Copyright © 2021 Wang et al. 2021 Wang et al. This content is distributed under the terms of the Creative Commons Attribution 4.0 International license.

Second, we measured the recovery rates of the five internal standards, five bacteria, and five fungi of different absolute abundances during genomic DNA extraction from a solid substrate (steamed sorghum) (Fig. 1B, part 2; see Materials and Methods). The results illustrate that the recovery rates of the internal standards, bacteria, and fungi were 70.02 to 83.90%, 71.87 to 87.10%, and 70.45 to 86.12%, respectively, with no significant differences (*P > *0.05 [Fig. S2]).

10.1128/mSystems.00964-20.2 FIG S2 Average recovery rates of internal standards, bacteria, and fungi in solid substrate (*n* = 5). Recovery rates of internal standards, bacteria, and fungi in ddH_2_O are 100% (control). The same letter indicates that there is no statistically significant difference (*P > *0.05). Download FIG S2, PDF file, 0.01 MB. Copyright © 2021 Wang et al. 2021 Wang et al. This content is distributed under the terms of the Creative Commons Attribution 4.0 International license.

Third, we calibrated the internal standards to eliminate the deviations between the quantitative equations of the internal standards, bacteria, and fungi, because we assumed that the internal standards cannot be completely equivalent to the microbes in the ecosystems. We built two staggered mock communities using 10 bacteria and fungi common in liquor fermentation. We then added five different absolute abundances of internal standards (approximately 10^4^, 10^5^, 10^6^, 10^7^, and 10^8^ copies g^−1^) to each mock community (Fig. 1B, part 3; see Materials and Methods). Then, we extracted the internal standards (plasmids) and microbial genomic DNA for quantification (see Materials and Methods). The quantitative equation established by internal standards was as follows:

$$y_{\text{CT-value}}=a\text{ × }x_{\text{internals}}+b$$

which was calibrated to

$$y_{\text{CT-value}}\text{ = }a\text{ × }c\text{ × }x_{\text{internals}}+b\text{ × }d$$

where *a* and *b* are the slope and intercept of the internal standards and microbes, respectively, *c* and *d* are the calibrated coefficients in the internal standards and microbes, respectively, *x*_internals_ is the concentration of the internal standards (log_10_ copies per milliliter) or microbes (log_10_ copies per milliliter), and *y*_CT-value_ is the cycle threshold value (CT-value) of qPCR results.

**FIG 2:**
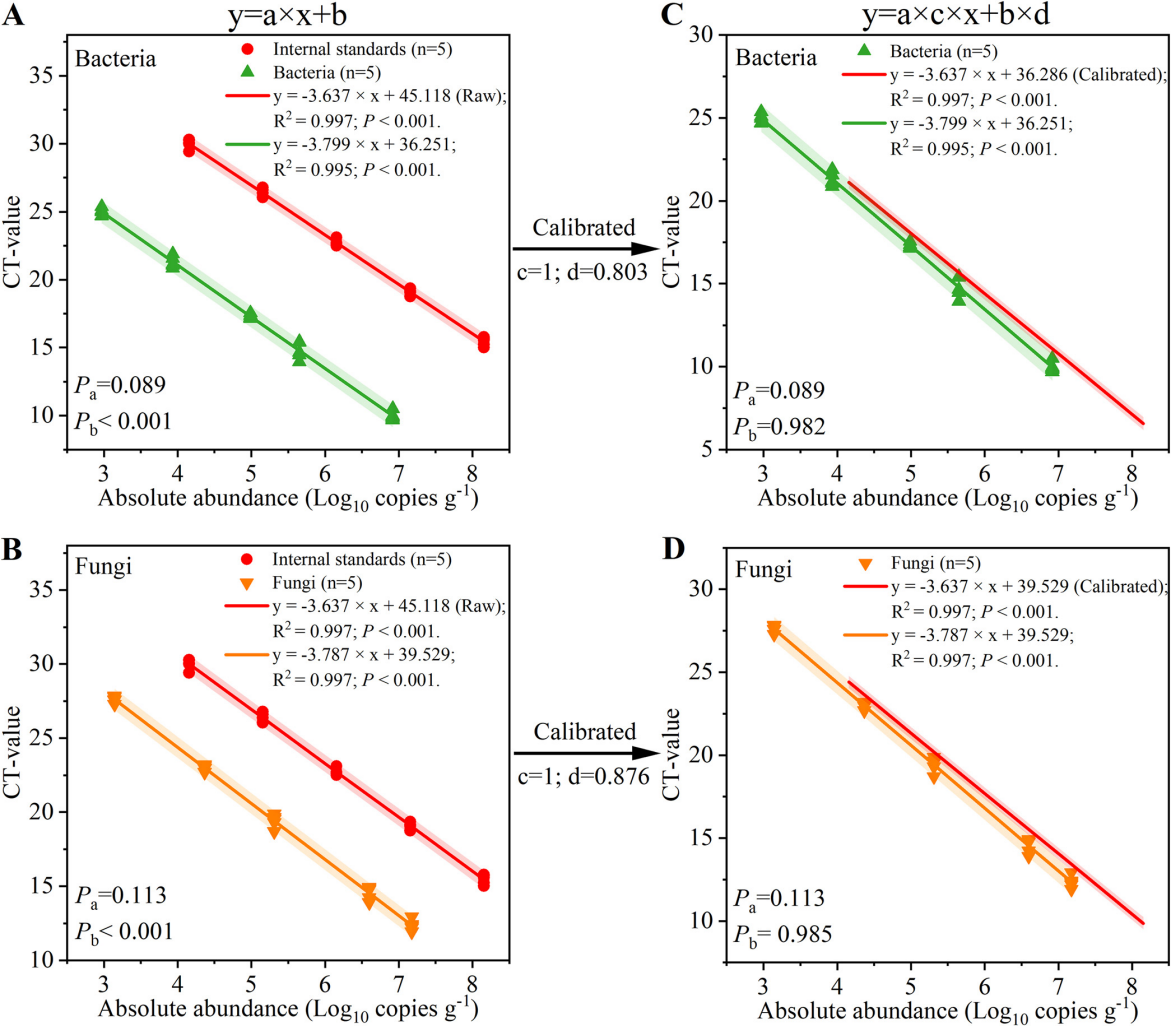
Calibration of the quantitative equations of the internal standards. (A) The equations of internal standards and bacteria before calibration. (B) The equations of internal standards and fungi before calibration. (C) The equation of internal standards calibrated by the equation of bacteria. (D) The equation of internal standards calibrated by the equation of fungi. *P_a_* and *P_b_* were calculated using one-way ANOVA.

An analysis of variance (ANOVA) of the slopes (variable *a* in the equation) and intercepts (variable *b* in the equation) of the quantitative equations of the internal standards, bacteria (*P_a_* = 0.089; *P_b_* < 0.001 [Fig. 2A]), and fungi (*P_a_* = 0.113; *P_b_* < 0.001 [Fig. 2B]) demonstrated that there were no significant differences (*P_a_* > 0.05) between the slopes, but there were significant differences (*P_b_* < 0.001) between the intercepts. Thus, we attempted to calibrate the quantitative equations of the internal standards based on the intercepts of bacteria and fungi. Due to the lack of a significant difference between the slopes (*P_a_* > 0.05), the slope calibrated coefficients were 1 (*c*_bacteria_ = 1; *c*_fungi_ = 1 [Fig. 2C and D]). The intercepts of the quantitative equations of the internal standards, bacteria, and fungi were 45.118 ± 1.316, 36.251 ± 0.534, and 39.529 ± 0.970, respectively (Fig. 2A and B). Therefore, the calibrated coefficients were 0.803 and 0.876 for bacteria and fungi, respectively (*d*_bacteria_ = 36.251/45.118 = 0.803; *d*_fungi_ = 39.529/45.118 = 0.876 [Fig. 2C and D]). When the calibrated equations were used, the deviations between the internal standards and microbes effectively disappeared (bacteria, *P_b_* = 0.982; fungi, *P_b_* = 0.985 [Fig. 2C and D]), producing excellent agreement between the results of the quantitative equations of the internal standards and microbes.

**FIG 3:**
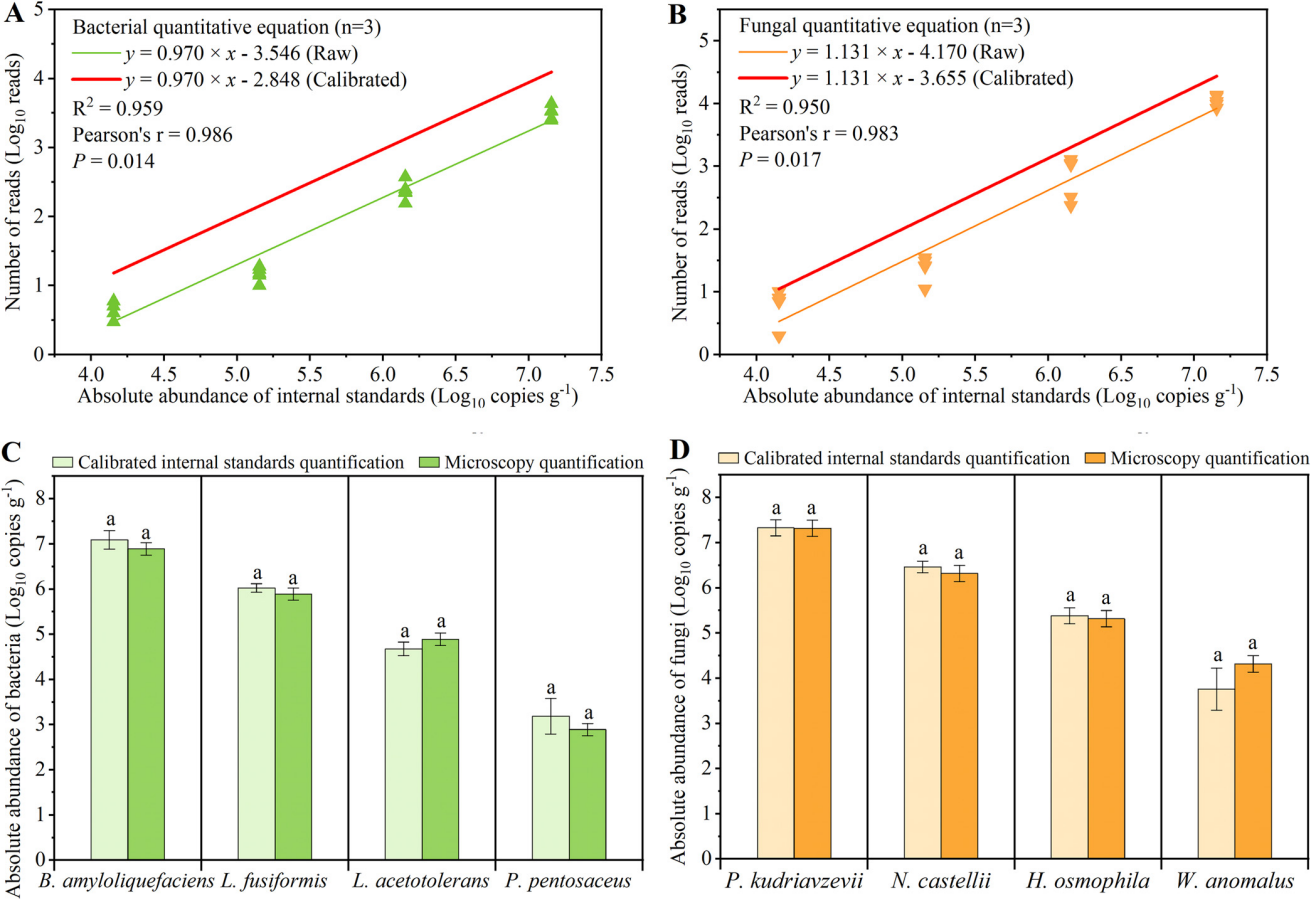
Detection of the microbial absolute abundances in the mock communities using GIS-AQ. (A) Average calibration equation of bacteria (*n* = 5). (B) Average calibration equation of fungi (*n* = 5). (C) Comparison of two absolute quantitative methods for bacteria. (D) Comparison of two absolute quantitative methods for fungi. The same letter (a) indicates no statistically significant differences (*P > *0.05).

Fourth, amplicon sequencing was used to sequence the genome samples extracted in the third step. We assumed that the deviation between the quantitative results of the internal standards and microbes in the third step would also exist between the results of amplicon sequencing. Therefore, the calibrated coefficients (*d*_bacteria_ = 0.803; *d*_fungi_ = 0.876) obtained in the third step were applied to the quantitative equations of amplicon sequencing (Fig. 3A and B) (see Materials and Methods). We used microscopy quantitation methods to validate the GIS-AQ method. Microscopy quantitation is the most accurate quantitative method because it can directly count the number of added individual microbes (15, 22). Therefore, the accuracy of the GIS-AQ method can be validated if there was no significant difference between the quantitative results by calibrated quantitative equations and microscopy quantitation. Based on the gradient of the internal standards and calibrated coefficients, we established calibrated quantitative equations for the bacteria and fungi (Fig. S3) in each sample.

10.1128/mSystems.00964-20.3 FIG S3 Quantification of bacteria and fungi in mock communities. Each mock community exhibited a unique quantitative equation. Download FIG S3, PDF file, 0.02 MB. Copyright © 2021 Wang et al. 2021 Wang et al. This content is distributed under the terms of the Creative Commons Attribution 4.0 International license.

Finally, we compared the different quantitative results (Fig. S4A and B) and found that there were no significant differences (*P > *0.05) between the quantitative results using the calibrated quantitative equations and the microscopy quantitation in the mock communities (Fig. 3C and D). These results suggest that the GIS-AQ method can accurately (*P*_internals versus microscopy_ > 0.05) quantify the microbial absolute abundances.

10.1128/mSystems.00964-20.4 FIG S4 Detection of the absolute abundances of bacteria (A) and fungi (B) in the mock communities using three absolute quantitative methods. The different letters (a and b) indicate statistically significant differences (*P < *0.05). Download FIG S4, PDF file, 0.01 MB. Copyright © 2021 Wang et al. 2021 Wang et al. This content is distributed under the terms of the Creative Commons Attribution 4.0 International license.

### Perturbation of quantification with single internal standards.

Before we applied the GIS-AQ method to solid-state fermentation, we sought to answer two questions. (i) In the amplification sequencing process, does the use of internal standards affect the relative abundance of microbes in solid-state fermentation? (ii) Does the use of a single internal standard perturb the absolute abundance?

To determine the effect of additional internal standards on the solid-state fermentation, we only added one internal standard to three independent solid fermentation samples with 0 copies g^−1^ (control), approximately 10^5^ copies g^−1^, and 10^8^ copies g^−1^ (Fig. S5A and B) (see Materials and Methods). To facilitate comparison between the samples, we analyzed the composition of the microbial communities after the internal standards were removed.

10.1128/mSystems.00964-20.5 FIG S5 Relative abundances of bacteria (A) and fungi (B) before removal of the internal standards. The concentrations of the internal standards are approximately 0 copies g^−1^, 10^5^ copies g^−1^, and 10^8^ copies g^−1^. Download FIG S5, PDF file, 0.01 MB. Copyright © 2021 Wang et al. 2021 Wang et al. This content is distributed under the terms of the Creative Commons Attribution 4.0 International license.

**FIG 4:**
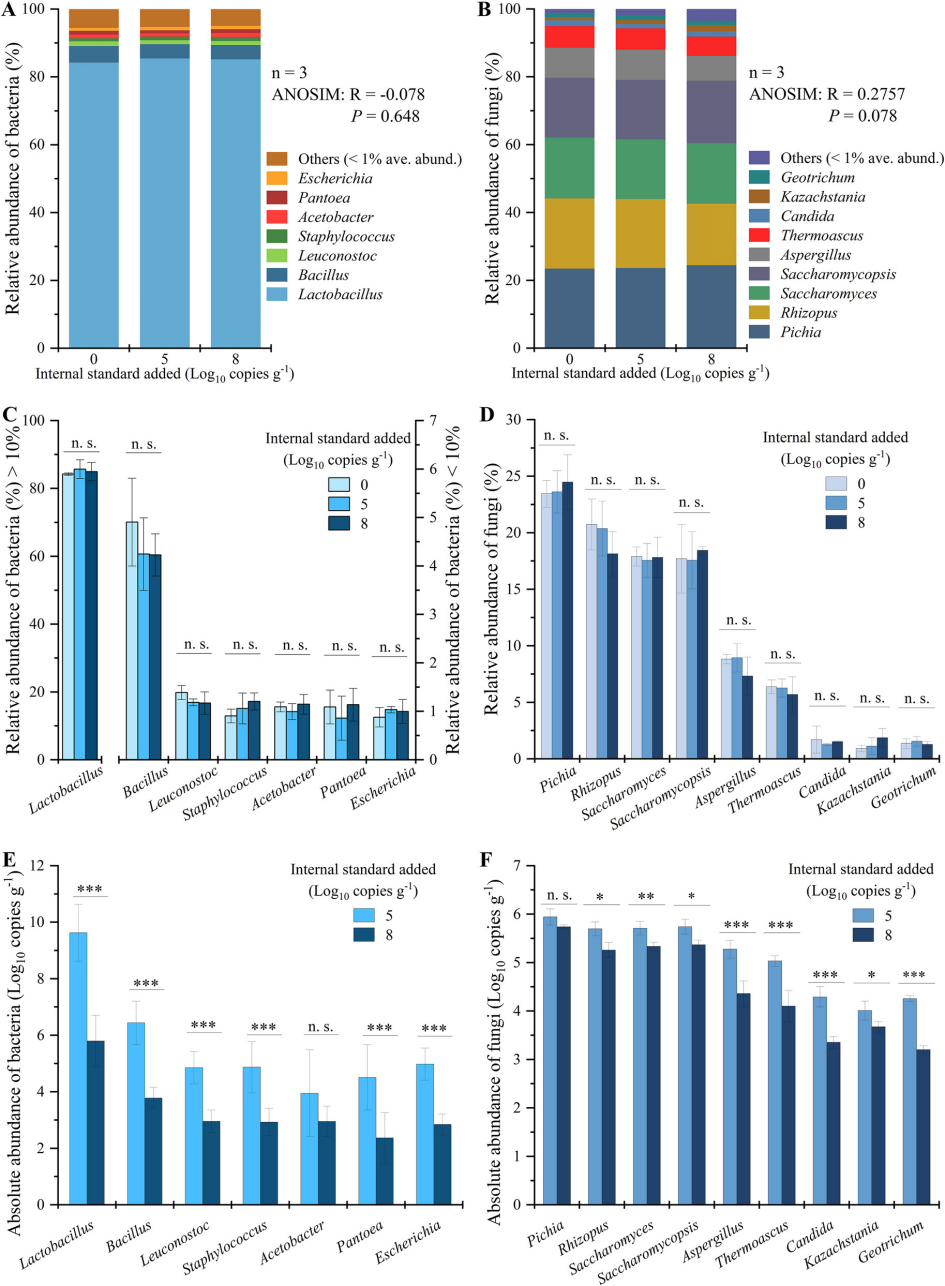
Perturbation of results with a single internal standard in solid-state fermentation samples (*n* = 3). (A and B) Relative abundances of bacteria (A) and fungi (B) after removing the internal standards. (C and D) Relative abundances of dominant bacteria (C) and fungi (D) (relative abundance ≥ 1%) in different samples (the concentrations of internal standards are approximately 0 copies g^−1^, 10^5^ copies g^−1^, and 10^8^ copies g^−1^). (E and F) Absolute quantitation comparison of dominant bacteria (E) and fungi (F) quantified by different concentrations of internal standards (approximately 10^5^ copies g^−1^ and 10^8^ copies g^−1^).

There were no significant differences between groups with different internal standards added in the bacterial communities (analysis of similarities [ANOSIM]: R = −0.078; *P = *0.648 [Fig. 4A]) and fungal communities (ANOSIM: *R* = 0.2757; *P = *0.078 [Fig. 4B]). Then, genera with an average relative abundance of ≥1% (dominant microbiota) were selected for analysis. There were no significant differences (*P > *0.05) in the relative abundances of the seven dominant bacteria (Fig. 4C) and nine dominant fungi (Fig. 4D) in the groups with different internal standards added. These results indicate that during the amplification sequencing process, the use of internal standards did not affect the relative abundances of microbes in solid-state fermentation.

The absolute abundances of bacteria and fungi were obtained based on the concentrations of the single internal standard (approximately 10^5^ copies g^−1^ and 10^8^ copies g^−1^). The absolute abundances of all bacteria were significantly different (*P < *0.05) between the two internal standards of different concentrations (except for *Acetobacter*) (Fig. 4E). The absolute abundances of all fungi were also significantly different (*P < *0.05) between the two internal standards of different concentrations (except for *Pichia*) (Fig. 4F). These results indicate that adopting gradient internal standards is necessary because of the quantitative perturbation that results from using a single internal standard.

### Detection of the absolute abundance of microbiota in solid-state fermentation samples using the GIS-AQ method.

To explore the difference between the relative and absolute abundances, the Chinese liquor solid-state fermentation (a type of ecosystem) (see Materials and Methods) was used as a model system for quantification. Microbial compositions were constructed for the solid-state fermentation samples from the first and second batches based on 16S rRNA and ITS sequences (Fig. S6A and B). The absolute quantitative equations of bacteria and fungi were obtained based on the calibrated quantitative equations of the internal standards (Fig. S7). Then, the absolute abundances of the communities in the fermentation were calculated based on the calibrated quantitative equations (Fig. S6C and D).

10.1128/mSystems.00964-20.6 FIG S6 Abundances of bacteria and fungi in the Chinese liquor solid-state fermentation (*n* = 3). (A) The relative abundances of bacteria. (B) The relative abundances of fungi. (C) The absolute abundances of bacteria. (D) The absolute abundances of fungi. Download FIG S6, PDF file, 0.01 MB. Copyright © 2021 Wang et al. 2021 Wang et al. This content is distributed under the terms of the Creative Commons Attribution 4.0 International license.

10.1128/mSystems.00964-20.7 FIG S7 Quantitative equations for the absolute abundance of bacteria and fungi in Chinese liquor solid-state fermentation. Download FIG S7, PDF file, 0.01 MB. Copyright © 2021 Wang et al. 2021 Wang et al. This content is distributed under the terms of the Creative Commons Attribution 4.0 International license.

**FIG 5:**
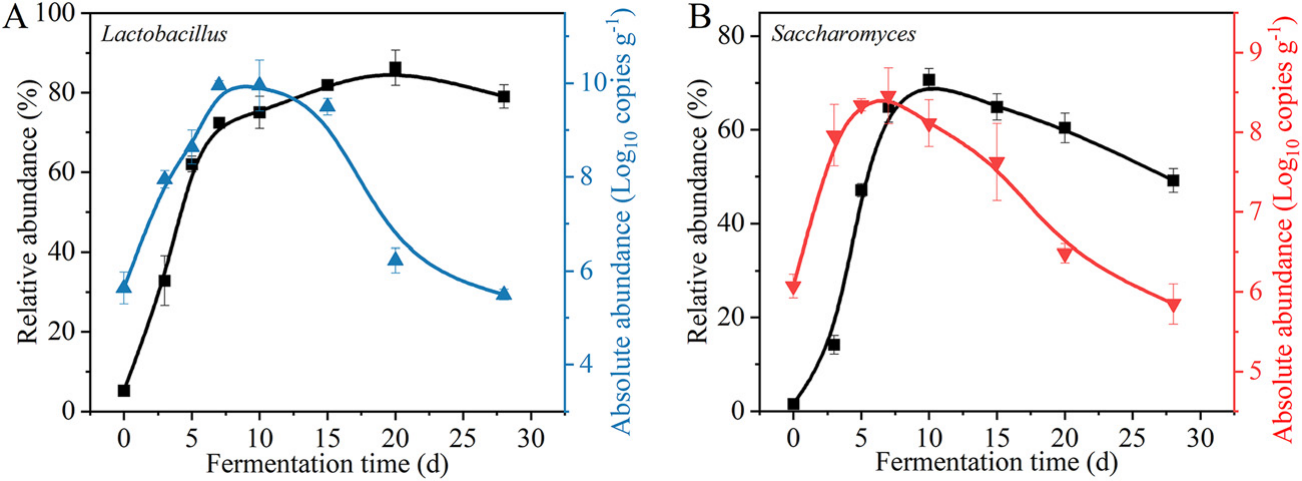
Abundances of *Lactobacillus* and *Saccharomyces* in the Chinese liquor solid-state fermentation (*n* = 3). (A) Relative and absolute abundances of *Lactobacillus*. (B) Relative and absolute abundances of *Saccharomyces*.

For bacteria, *Lactobacillus* (absolute dominant genus; ≥10% average abundance) was selected as an example to illustrate the difference between relative and absolute abundance. The relative abundance of *Lactobacillus* gradually increased until day 20 of fermentation, while the absolute abundance reached its peak on day 7 (Fig. 5A). For fungi, *Saccharomyces* (absolute dominant genus) was selected as an example to illustrate the difference between relative and absolute abundance. The relative abundance of *Saccharomyces* gradually increased until day 10 of fermentation, while the absolute abundance reached its peak on day 7 (Fig. 5B).

In summary, the results obtained from the relative and absolute abundances are different. The relative abundance is more suitable for the study of the different compositions of communities in one sample. The absolute abundance can be used to study the difference in communities between different samples, and the GIS-AQ method allows this to be done swiftly.

## DISCUSSION

### Characteristics of different quantitative methods.

We established and implemented a gradient internal standard absolute quantification (GIS-AQ) method to determine the absolute abundances of bacteria and fungi. This method establishes a reference and extends existing quantitative methods for microbial communities. Every method has its advantages and disadvantages; therefore, we compared the GIS-AQ method with existing quantitative methods to provide context and allow the reader to choose the most appropriate method for their respective research question. To resolve the shortcomings of traditional amplicon sequencing, various methods have been proposed to assess the absolute abundance of microbiota, such as flow cytometry (11, 16, 17), qPCR (17), fluorescence *in situ* hybridization coupled with catalyzed reporter deposition (CARD-FISH) (23), and spike-in (internal standard) (12, 14, 15, 22, 24–26). However, determining the absolute abundance using these methods remains challenging. For example, combining amplicon sequencing and flow cytometry can accurately quantify the number of cells in liquid samples. However, it is mainly suitable for specific labeled cells in the liquid samples. In the case of qPCR, the external standard used for qPCR quantification seldom has the same recovery rate as the ecosystem. The CARD-FISH method is complicated and time-consuming to operate. The internal standard approach is the most widely used quantitative method. For example, some researchers designed three internal standards (plasmids) for prokaryotic 16S rRNA, eukaryotic 18S rRNA, and fungal ITS in soil samples. To realize the microbial quantification, three internal standards were added before DNA extraction and repeated DNA extraction and sequencing, changing the concentration of internal standards in each repetition and establishing a quantitative equation, of the same soil sample (14). Researchers designed a synthetic full-length 16S rRNA gene spike-in standard for prokaryotic 16S rRNA in soil samples, and they also used the same method as before to achieve the quantification of prokaryotic 16S rRNA (26). Compared with the existing internal standard quantitative methods, the GIS-AQ method can be described as follows. (i) We constructed a gradient internal standards group, and one round of amplicon sequencing can achieve the determination of different concentrations of internal standards. The GIS-AQ method is also faster and more economical than performing amplification sequencing multiple times to establish a quantitative equation. (ii) We designed the primer information of bacteria and fungi on the same internal standard sequence, which facilitates the quantification of bacteria and fungi at the same time. (iii) Based on the mock communities, the quantitative equations of the internal standards were calibrated to eliminate the potential deviations between the internal standards and communities in the process of genome DNA extraction. Therefore, we believe that the GIS-AQ method has advantages for quantifying microbial communities.

### Application of the absolute abundance data.

The relative abundance of amplicon sequencing is based on the normalization or transformation of the total read number (27). Relative abundance is suitable for statistical analysis of the same samples or samples that have similar communities, such as natural soils (28) and healthy guts (29). For samples with large differences in communities, such as fertilizing soils (25) and patients’ guts, the relative abundance may easily generate information that is contrary to the actual situation (11). Therefore, at present, the analysis method of combining relative and absolute abundances is primarily used for samples with large differences in microbial communities. Specific manifestations are as follows. (i) When characterizing the changes in communities, the absolute abundance is more convincing than the relative abundance because it shows the dynamic changes in communities between different samples (such as time series samples) that cannot be seen by traditional relative abundance (11). This application represents the most important use of absolute abundance (14). (ii) Regarding the replacement of relative abundance data with absolute abundance data in traditional statistical analysis, at present, no systematic comparative studies that have shown that absolute abundance data can be used in traditional statistical analysis methods are known; however, some researchers have attempted substitution in different statistical methods, such as principal-component analysis (25), network analysis (17), and cluster patterns (heat map) (15). The scope of the application of absolute abundance in the field of microbial quantification still requires further research to support and verify current results. Thus, absolute abundance is a crucial challenge for future microbiota variance studies (24).

### Applicability of the GIS-AQ method.

The GIS-AQ method established in this study provides a new reference for the field of microbial quantification. As the microbial abundances, species, and substrates are different in various research fields, the GIS-AQ method can be applied to different research fields after appropriate adjustments. These adjustments mainly reflect the design of the internal standards, mock communities, and substrates. (i) The parameters of the internal standards should be designed according to the general primer, GC content, and the sequence length corresponding to the amplification regions. For example, when the amplification region is V4 (30), V4-V5 (31), or ITS1 (32), it is necessary to change the primer of the internal standards used. (ii) The mock communities and substrates should be the same or similar to the object of study because the quantitative principle of internal standards is to use the quantitative equations of the internal standards to quantify the absolute abundance of the microbes. At present, it is unknown whether differences exist between the internal standards and microbes in the process of genome extraction and amplification; however, the problem can be solved using mock communities and substrates. Therefore, when studying the oceans (33), the mock communities should have microbes with different abundances in the oceans, and the mock substrates chosen should be a sterile isotonic saline solution with a seawater composition or sterilized seawater.

### Matters requiring attention in the use of the GIS-AQ method.

The amount of manual labor required by the GIS-AQ method is larger than for existing quantitative research using internal standards (25). This is mainly due to the construction of the mock communities and substrates. As we assume that the internal standards cannot be completely equivalent to the microbes in the natural systems, it is necessary to eliminate the deviations between the internal standards and microbial quantitative equations. Theoretically, different mock communities and substrates need to be constructed for various research ecosystems. However, if there is no change in the ecosystem, the mock communities and substrate need only be constructed once; therefore, the extra manual labor required is limited. In the Chinese liquor solid-state fermentation ecosystem, the detection line of GIS-AQ method is 10^4^ to 10^5^ copies g^−1^, which is a very low detection line compared with that for the dominant microbes (10^8^ to 10^9^ copies g^−1^) (21, 34). Furthermore, the concentration ranges of the internal standards can be adjusted according to the microbial concentration ranges in different ecosystems, such as soils (10^5^ to 10^9^ copies g^−1^) (25) and guts (10^7^ to 10^11^ copies g^−1^) (17). For the use of the GIS-AQ method, the following points should be noted. (i) There should be uniformity between the internal standards, because different internal standards need to be used to represent various concentrations. (ii) The concentration gradients of microbes in mock and natural communities should be similar. (iii) When calibrating the quantitative equations, the difference in the calibration coefficients between bacteria and fungi cannot be ignored.

In summary, we developed a GIS-AQ method for bacteria and fungi. The GIS-AQ method expands upon traditional amplicon sequencing based on relative abundance. Based on the design principle, after proper modification, the GIS-AQ method is compatible with any application using any amplification region (such as V3, V4, V3-4, ITS1, and ITS2) and is thus applicable in a broad range of ecosystems, including soil, rhizospheres, and food. Therefore, the GIS-AQ method is a valuable addition to microbiology research.

## MATERIALS AND METHODS

### Design and construction of internal standard sequences.

The internal standard sequences (ISS) were designed based on three key elements. (i) The internal standard sequences had the same universal primer binding sites (PBSs) as those of the bacteria and fungi in the amplicon sequencing samples. (ii) The synthetic stuffer sequences had a similar length and GC% as that of the microbes in the solid-state fermentation. (iii) The specific PBSs were used to recognize the internal standard sequences (Fig. 1).

First, the primers 336F/806R of the 16S rRNA V3-V4 region (35) and ITS3/ITS4 of the ITS2 region (36) were used as universal primers for bacteria and fungi for the internal standards. Second, based on previous research (21), we selected the average length of amplified fragments and GC content of the 10 microbes (five bacteria and five fungi [Table S1]) with the highest relative abundances in the solid-state fermentation process as the references to construct the random sequences (14). There was a set of random sequences (Table 1) that satisfied the following criteria: uniform GC content (±1%), no homopolymers of >10 bp, no repeats of >16 bp (as determined by BLAST), and no self-complementary regions of >10 bp (26). In addition, the optimized set of artificial sequences shared negligible identity with sequences in the National Center for Biotechnology Information (NCBI) database. Third, we designed specific primers for internal standards sequences based on the NCBI Primer-BLAST (Table S2). The specific primers satisfied the following criteria: similar length, similar melting temperature (*T_m_*) values, similar GC contents, no homopolymers of >3 bp, and no self-complementary regions of >5 bp (26). The designed primers were used as upstream and downstream primers, and BLAST was performed in the NCBI database, which demonstrated negligible identification with known sequences in the NCBI nucleotide collection (nr/nt) database (web-BLAST performed in May 2019). The specificity of these primers was demonstrated by a PCR cross-validation. The PCR conditions were as described in a previous study (37).

10.1128/mSystems.00964-20.8 TABLE S1 Highest relative abundance of bacteria and fungi in Chinese liquor solid-state fermentation process. Download Table S1, XLSX file, 0.02 MB. Copyright © 2021 Wang et al. 2021 Wang et al. This content is distributed under the terms of the Creative Commons Attribution 4.0 International license.

10.1128/mSystems.00964-20.9 TABLE S2 Specific primers used in this study. The specificity of these primers was demonstrated by PCR cross-validation. Download Table S2, XLSX file, 0.02 MB. Copyright © 2021 Wang et al. 2021 Wang et al. This content is distributed under the terms of the Creative Commons Attribution 4.0 International license.

Complete internal standards sequences are provided in Table 1. Full-length internal standard sequences (472 bp) were chemically synthesized and inserted into the pUC-57 plasmid cloning vector by Sangon Biotech (Shanghai, China). Plasmid cloning vectors with internal standard sequence inserts were transformed into Escherichia coli TOP10 competent cells [genotype: F^−^ *mcrA* Δ(*mrr-hsdRMS-mcrBC*) φ80 *lacZ*ΔM15Δ lacX74 *recA1 ara*Δ139Δ(*ara-leu*)7697 *galU galK rpsL* (Str^r^)*endA1 nupG*] (Sangon Biotech) as per the manufacturer’s instructions. Internal standard DNA was extracted from overnight liquid cultures using a SanPrep spin column and collection tube (Sangon Biotech) according to the manufacturer’s instructions. The internal standard DNA concentrations were measured using a PicoGreen double-stranded DNA (dsDNA) assay kit (Invitrogen Life Technologies, Grand Island, NY) (26), and the internal standard concentration was calculated as

$$C_{\text{internal }}{\text{(copies ml}}^{\text{-1}}\text{) = }\frac{{\text{m}}_{\text{DNA}}\left({\text{ng ml}}^{\text{-1}}\right)\text{ × 6}{\text{.022 × 10}}^{\text{23}}\left({\text{copies mol}}^{\text{-1}}\right)}{\text{DNA length }\left(\text{bp}\right){\text{ × 10}}^{\text{9}}\left({\text{ng g}}^{\text{-1}}\right){\text{ × 660 (Da mol}}^{\text{-1}}\text{)}}$$

where *C*_internal_ is the internal standards concentration, and m_DNA_ is the mass of the internal standards. In our study, five internal standards (IS1 to IS5) were stored at –80°C for future processing at a concentration of 1 μg ml^−1^.

### Strains.

The microbes used in the experiment were isolated from the Chinese liquor solid-state fermentation process: Bacillus amyloliquefaciens LBM 10008, Lysinibacillus fusiformis LBM 10019, Lactobacillus acetotolerans LBM 10005, Weissella paramesenteroides LBM 10007, Pediococcus pentosaceus LBM 10024, Pichia kudriavzevii LBM 20017, Naumovozyma castellii LBM 20004, Hanseniaspora osmophila LBM 20013, Wickerhamomyces anomalus LBM 20006, and Saccharomyces cerevisiae LBM 20001. Using the internal standard-specific primer design method, we constructed specific primers (Table S2) for the microbes. The specificity of these primers was demonstrated using PCR cross-validation.

### qPCR.

The qPCR was performed on a real-time PCR system (Applied Biosystems, Foster City, CA) with a mixture of 0.4 μl (20 μM) of primers, 10.0 μl of SYBR green supermix (SYBR Premix *Ex Taq* II; TaKaRa, Shanghai, China), 1.0 μl of DNA templates, and 8.2 μl of double-distilled water (ddH_2_O) (38). The PCR program was as follows: preheating at 98°C for 10 min, 40 cycles at 98°C for 30 s and 60°C for 30 s, and an increase of 0.5°C every 5 s from 72°C to 95°C for the melting-curve analysis to confirm the amplification specificity (39).

### The calibrated coefficients.

The recovery rates of internal standards and microbes differed in the genome extraction (14). To calibrate the differences in the recovery rate, we designed the following experiments.

First, we diluted all five internal standards (IS1 to IS5) according to the gradient of approximately 10^4^, 10^5^, 10^6^, 10^7^, and 10^8^ copies ml^−1^. Then, five internal standards of the same concentration were mixed to obtain the internal standard concentration gradient. qPCR was performed on the mixed liquid to determine whether there was an amplification preference among the various internal standards. Second, genomic DNA was extracted from the five groups of mixed liquid using the easy nucleic acid isolation (E.Z.N.A.) soil DNA kit (Omega Bio-Tek, Inc., Norcross, GA) according to the manufacturer’s instructions to determine the internal standard recovery rate. Third, we used qPCR to establish the melting curves for five internal standards, five bacteria, and five fungi to discern the differences between their recovery rates. Finally, according to the microbe recovery rates, the quantitative equation established by internal standards, provided in Results, was determined.

### Mock communities.

To demonstrate the GIS-AQ method, we used mock communities to mimic the taxonomic diversity of the microbes in the solid-state fermentation process. Based on previous research (21), bacteria and fungi found to be dominant in the solid-state fermentation process of Chinese liquor fermentation were used as mock communities. *B. amyloliquefaciens* and *L. fusiformis* were incubated for 24 h at 37°C and 200 rpm in a Luria-Bertani sterile liquid medium (Sangon Biotech, Shanghai, China). *L. acetotolerans*, *W. paramesenteroides*, and P. pentosaceus were incubated for 48 h at 37°C in de Man-Rogosa-Sharpe medium (Oxoid Ltd., Hants, UK). *P. kudriavzevii*, N. castellii, *H. osmophila*, *W. anomalus*, and S. cerevisiae were incubated for 48 h at 30°C in a yeast extract-peptone-dextrose medium (Sangon Biotech).

After incubation, the 10 cultured bacteria and fungi were eluted with phosphate-buffered saline (0.01 M, pH 7.2) at 4,000 rpm for 20 min (5804 R centrifuge; Eppendorf, Hamburg, Germany) three times. Then, the microbes and internal standards were added to the sterile sorghum substrates after microscopy quantification. The sterile sorghum substrates were preprepared via autoclaving sorghum at 121°C for 20 min to simulate the substrates in the Chinese liquor solid-state fermentation process (21). The added concentrations of microbes were as follows: approximately 10^8^ copies g^−1^ of *B. amyloliquefaciens*, approximately 10^7^ copies g^−1^ of *L. fusiformis*, approximately 10^6^ copies g^−1^ of *L. acetotolerans*, approximately 10^5^ copies g^−1^ of *W. paramesenteroides*, approximately 10^4^ copies g^−1^ of P. pentosaceus, approximately 10^8^ copies g^−1^ of *P. kudriavzevii*, approximately 10^7^ copies g^−1^ of N. castellii, approximately 10^6^ copies g^−1^ of *H. osmophila*, approximately 10^5^ copies g^−1^ of *W. anomalus*, and approximately 10^4^ copies g^−1^ of S. cerevisiae. The added concentrations of internal standards were as follows: approximately 10^8^ copies g^−1^ of IS1, approximately 10^7^ copies g^−1^ of IS2, approximately 10^6^ copies g^−1^ of IS3, approximately 10^5^ copies g^−1^ of IS4, and approximately 10^4^ copies g^−1^ of IS5. Next, the sorghum substrates containing different absolute abundances of bacteria, fungi, and internal standards were uniformly mixed (Table S3). We performed five independent preparations for the mock communities.

10.1128/mSystems.00964-20.10 TABLE S3 Information for bacterial and fungal raw sequence data in the DDBJ database. Download Table S3, XLSX file, 0.02 MB. Copyright © 2021 Wang et al. 2021 Wang et al. This content is distributed under the terms of the Creative Commons Attribution 4.0 International license.

### Sample collection in natural solid-state fermentation.

For the solid-state liquor fermentation, steamed grains were mixed with the starter at a ratio of 9:1 (wt/wt) and fermented in sealed jars for 28 days. Subsequently, the fermented grains were distilled to obtain the liquor. We collected a total of 24 samples (100 g per sample) from 3 jars (three independent preparations) in the layer center (0.5 m deep) on days 0, 3, 5, 7, 10, 15, 20, and 28 (Table S3). All samples were stored at −80°C for further DNA extraction.

### Single internal standard perturbation experiment preparation.

To calibrate the influence of different concentrations of the internal standards on the results, we designed a single internal standard perturbation experiment. The perturbation experiment was designed based on the samples from day 10 of fermentation. Previous studies have demonstrated that the composition of the microbiota is stable and representative on day 10 of fermentation (21). We divided the same substrates (on day 10) into three parts (10 g each) and then added the internal standards (IS1) at 0 copies g^−1^ (control, equivalent ddH_2_O), 10^5^ copies g^−1^, and 10^8^ copies g^−1^ (Table S3) and mixed evenly. All samples were stored at −80°C for further DNA extraction. We conducted three independent preparations for all perturbation experiments.

### DNA extraction, qualification, and sequencing analysis.

Five-gram samples from the mock communities and natural fermentation were used to extract the total genomic DNA using an E.Z.N.A. soil DNA kit (Omega Bio-Tek, Norcross) according to the manufacturer’s instructions. The V3-V4 region of the 16S rRNA gene was amplified using the universal primers 336F (5′-GTACTCCTACGGGAGGCAGCA-3′) and 806R (5′-GTGGACTACHVGGGTWTCTAAT-3′) (35). For the fungi, the ITS2 region was amplified using the primers ITS3 (5′-GCATCGATGAAGAACGCAGC-3′) and ITS4 (5′-TCCTCCGCTTATTGATATGC-3′) (36). A unique 8-nucleotide barcode sequence was added to the primers to differentiate between the samples. PCR mixtures contained 2.5 μl of Pyrobest buffer (10×), 2 μl of deoxynucleoside triphosphates (dNTPs; 2.5 mM), 1 μl of each primer (10 μM), 0.4 U of Pyrobest DNA polymerase (TaKaRa Holdings, Inc., Kusatsu, Japan), 15 ng of template DNA, and ddH_2_O up to 25 μl. Amplification was performed as described previously (21). Amplicons were pooled in equimolar quantities and subjected to high-throughput sequencing using MiSeq sequencing for 2 × 300-bp paired-end sequencing (Illumina, San Diego, CA) (21).

### Sequence processing.

All raw sequences were processed using Quantitative Insights into Microbial Ecology (QIIME) (version 1.8) (40). High-quality sequences were obtained by removing sequences with >2 ambiguous bases, >10 homopolymers, primer mismatches, average quality scores of <20, and lengths (excluding the barcode region or primer) of <50 bp (21). Using USEARCH (version 10), chimeras were removed (41). UCLUST (version 1.2.22) clustered the trimmed sequences into operational taxonomic units (OTUs) with 97% sequence similarity (42). The Central Bureau of Fungal Cultures database (CBS-KNAW, www.wi.knaw.nl, a public database of fungal sequences), EzBioCloud database (www.ezbiocloud.net, a public database of bacterial sequences), and internal standard sequence information (Table S2) were used to align bacterial 16S rRNA gene, fungal ITS2 region, and internal standard sequences, respectively (21).

### Absolute quantitation.

To detect the absolute abundance of the microbes in the mock communities and ecosystems, we used gradient internal standards to build a linear equation between the concentration (log_10_ copies per gram) and the number of reads (log_10_ reads) of internal standards. The quantitative equation is as follows:

$$y_{\text{reads}}\text{ = }a\text{ × }x_{\text{internals}}+b$$

which was calibrated to

$$y_{\text{reads }}\text{= }a\text{ × }c\text{ × }x_{\text{internals}}+b\text{ × }d$$

where *a* and *b* are the slope and intercept of the internal standards and microbes, respectively, *c* and *d* are the calibrated coefficients of the internal standards and microbes, respectively, and *x*_internals_ is the concentration of internal standards (log_10_ copies per gram).

Based on the above equations, absolute abundances representing the microbial copy number (*x*_internals_) in the mock and natural samples were calculated based on the number of reads (*y*_reads_) in the amplicon sequencing.

### Statistical analysis.

Diversity and statistical analyses were conducted using XLSTAT (version 19.02.42992). The significant difference between recovery rates, slopes and intercepts were calculated using one-way ANOVA using SPSS Statistics (version 22). The Shannon index and Chao1 estimator were calculated using QIIME (version 1.8) (43, 44). ANOSIM was used to test differences between samples based on the Bray-Curtis distance matrices (45).

### Data availability.

The bacterial and fungal raw sequence data were deposited in the DNA Data Bank of Japan (DDBJ) database under the accession numbers DRA010282 and DRA010283. The mock community cultures are available by request for noncommercial purposes.
