# Supplementary figures and images for: Gradient Internal Standard Method for Absolute Quantification of Microbial Amplicon Sequencing Data
Source: mSystems. 2021 Jan 12;6(1):e00964-20. doi: 10.1128/mSystems.00964-20 (PMC7901480; doi:10.1128/mSystems.00964-20)

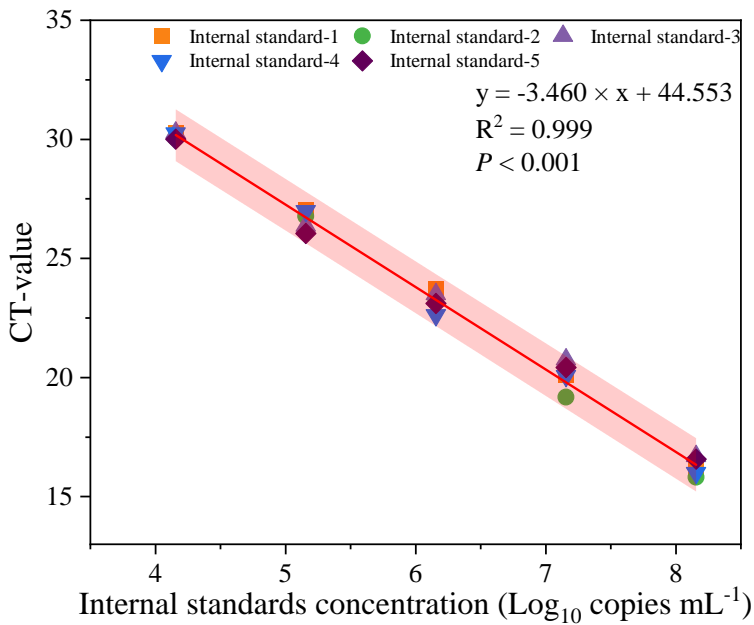

Supplement: FIG S1 [file mSystems.00964-20_sf001.pdf]

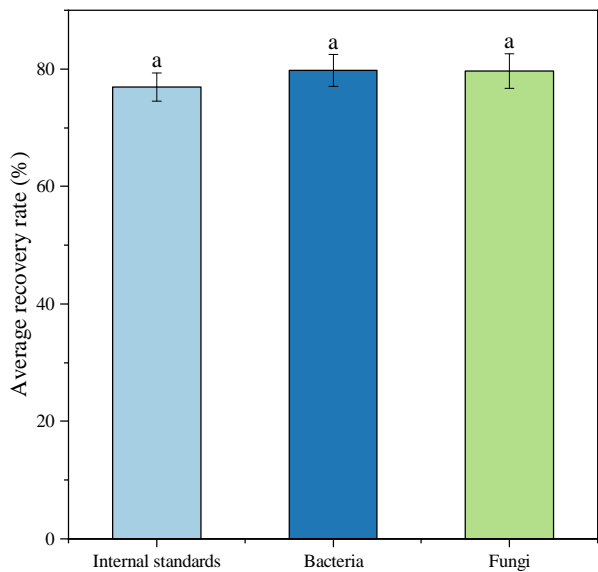

Supplement: FIG S2 [file mSystems.00964-20_sf002.pdf]

## Bacteria

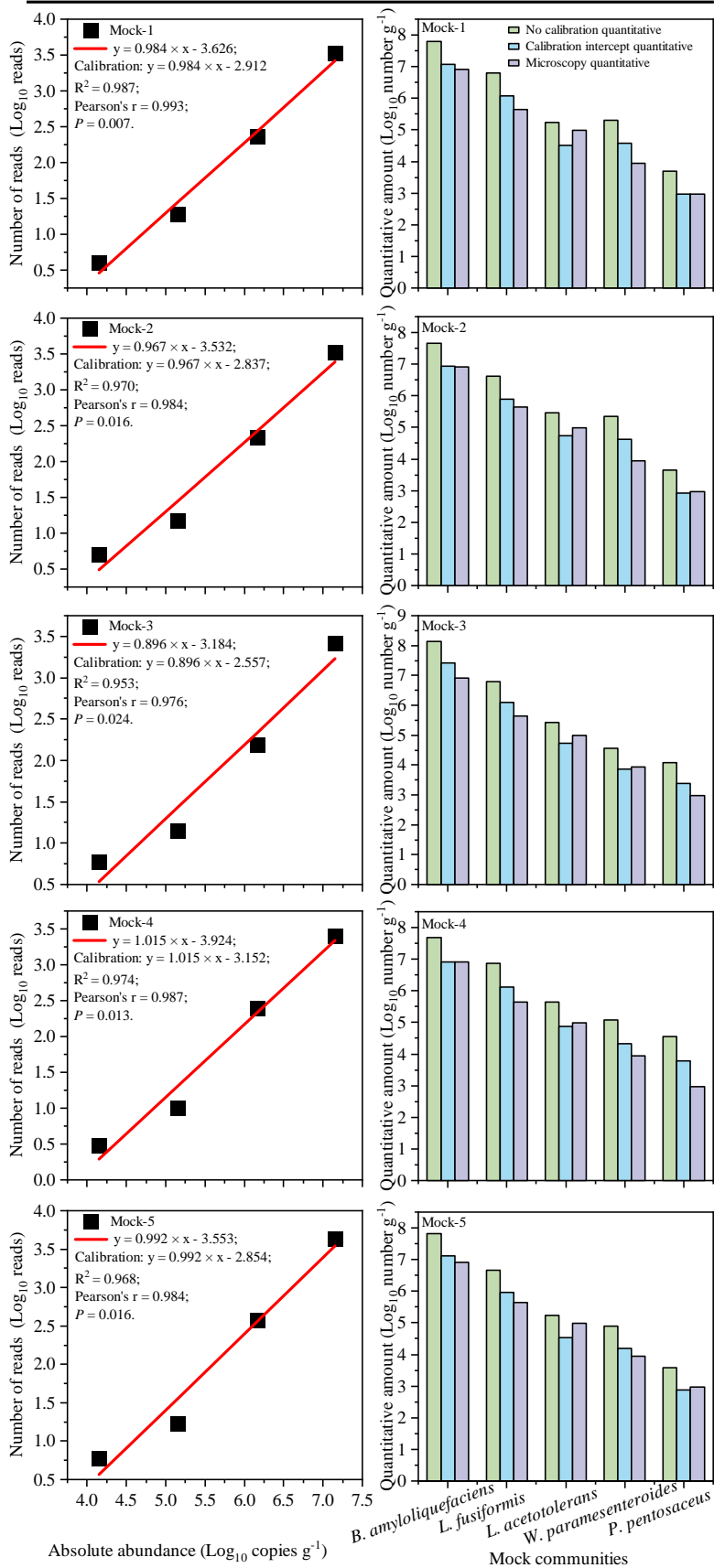

## Fungi

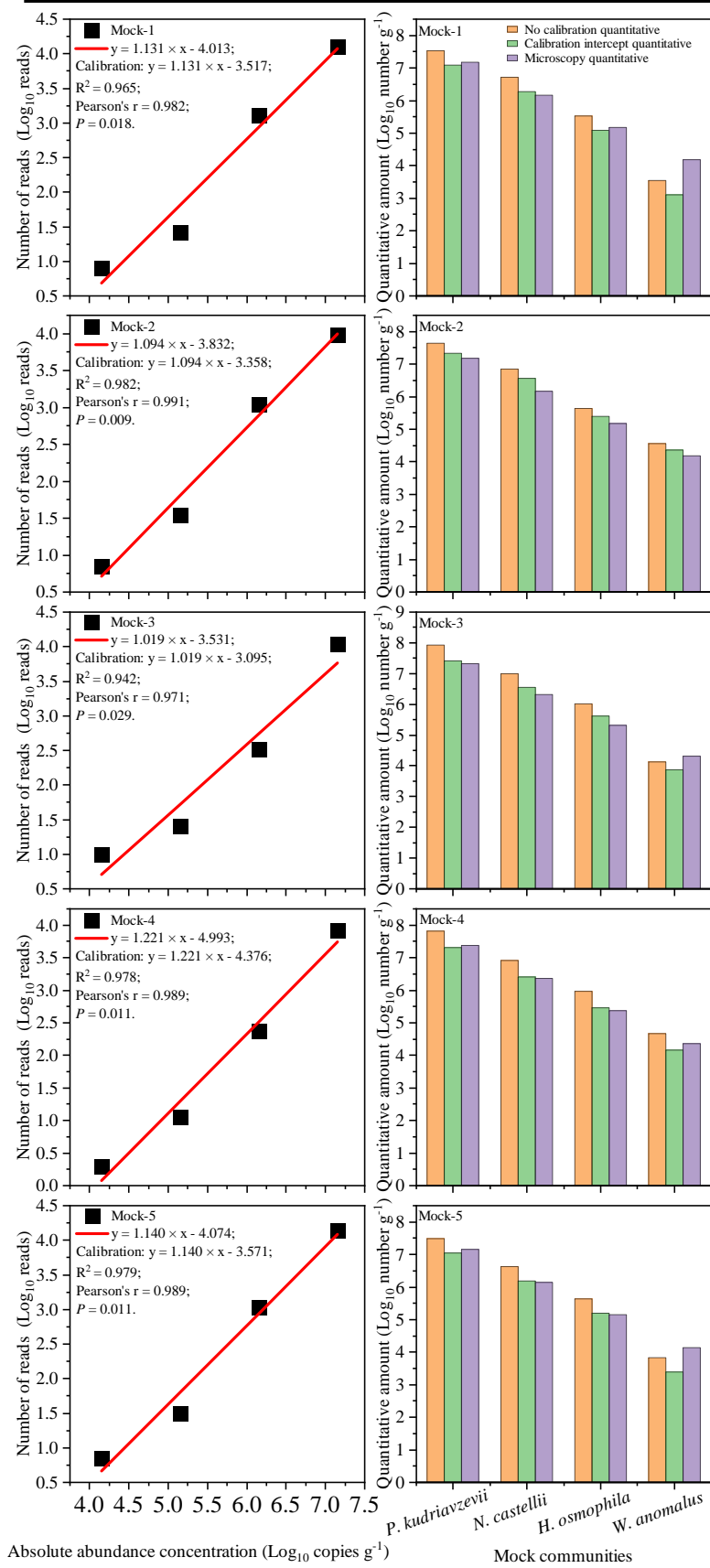

Supplement: FIG S3 [file mSystems.00964-20_sf003.pdf]

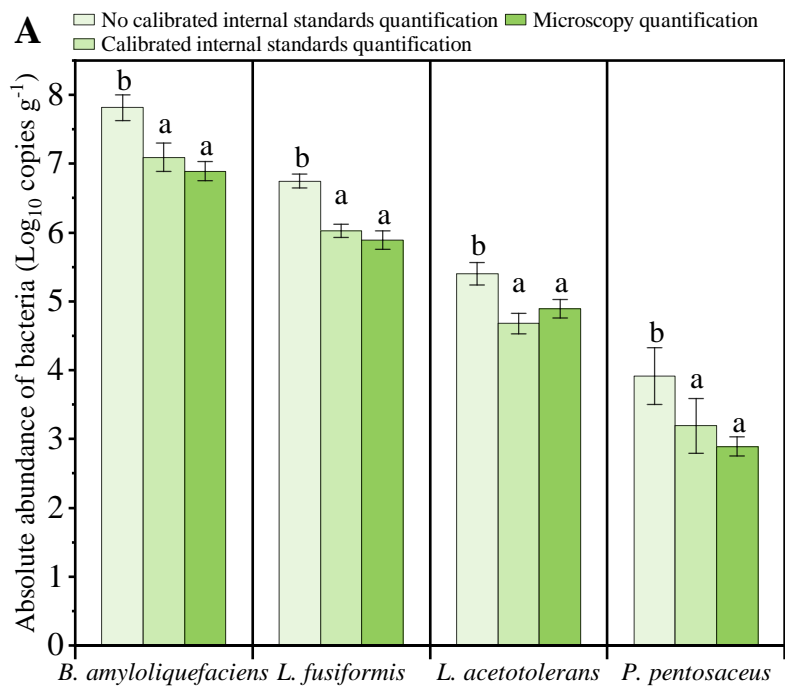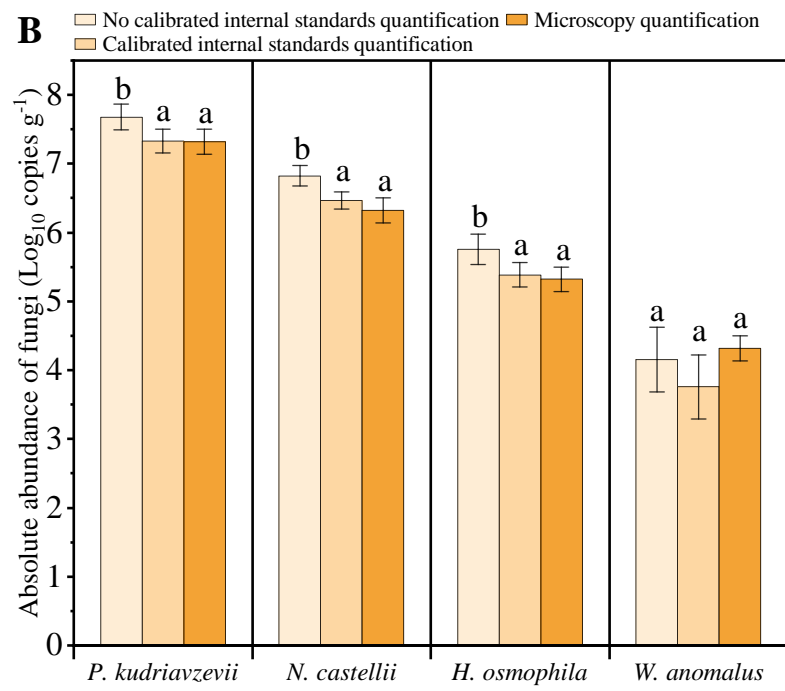

Supplement: FIG S4 [file mSystems.00964-20_sf004.pdf]

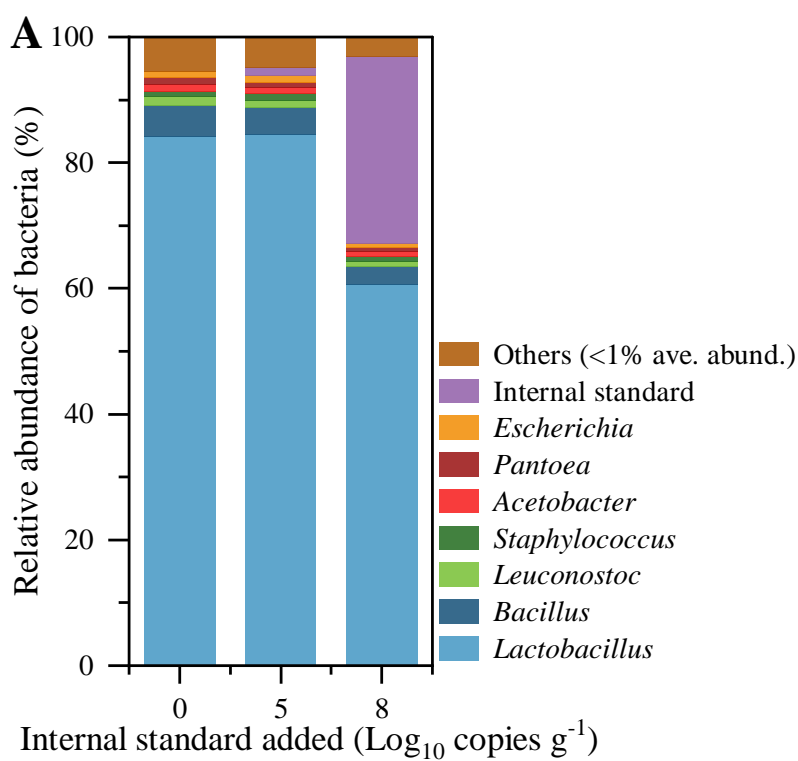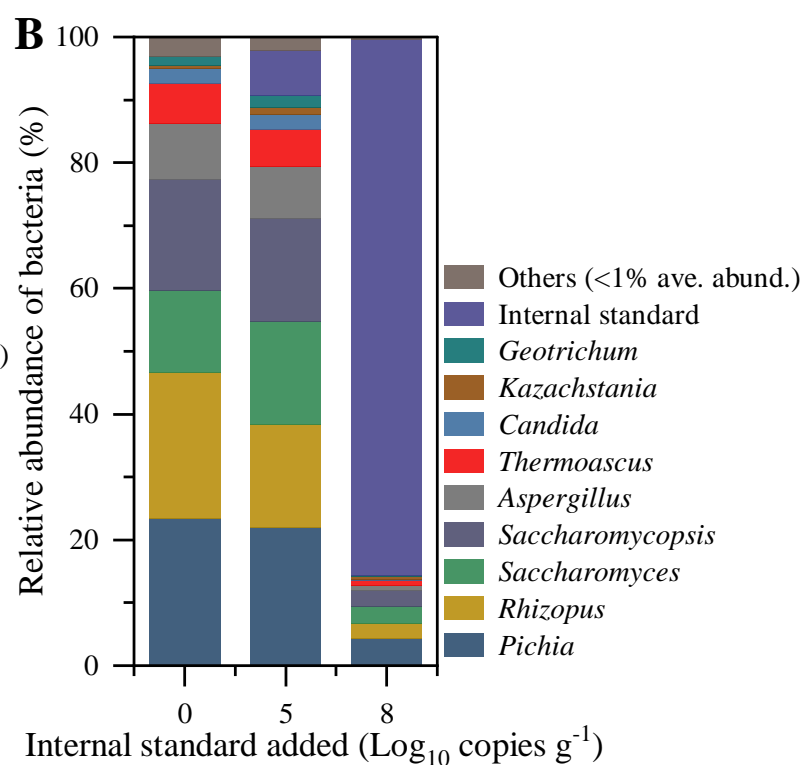

Supplement: FIG S5 [file mSystems.00964-20_sf005.pdf]

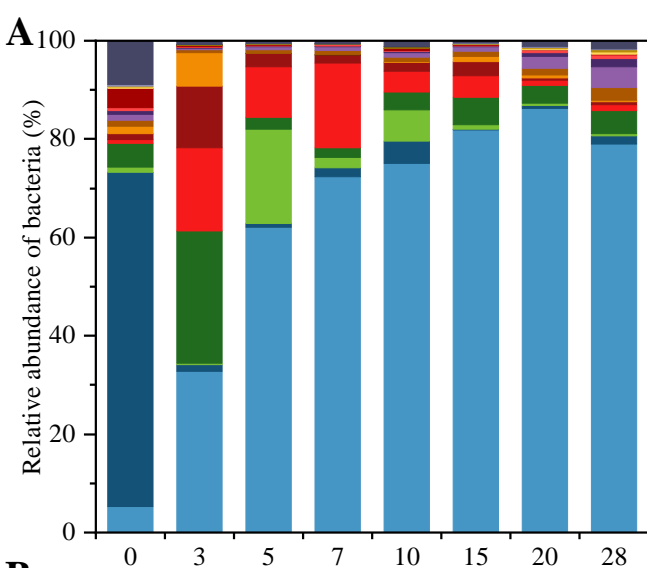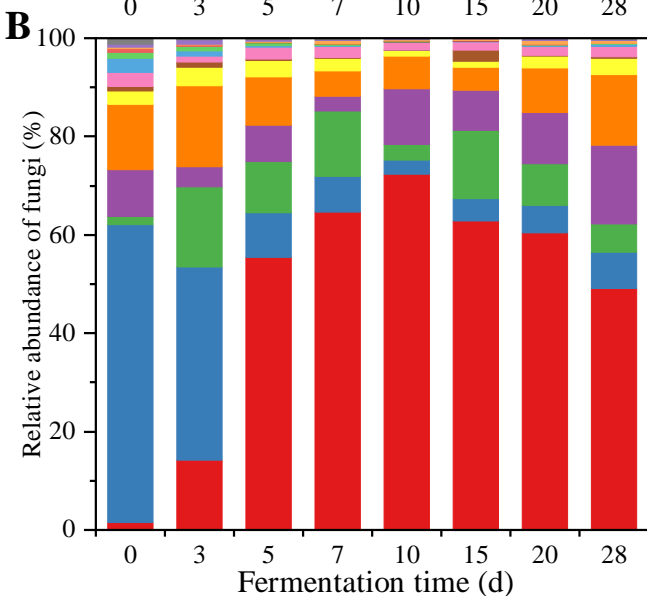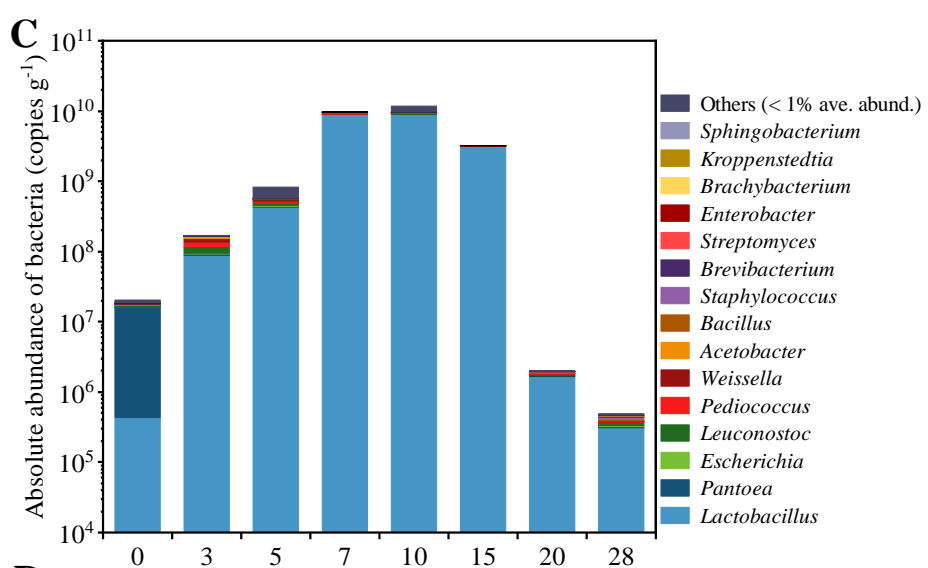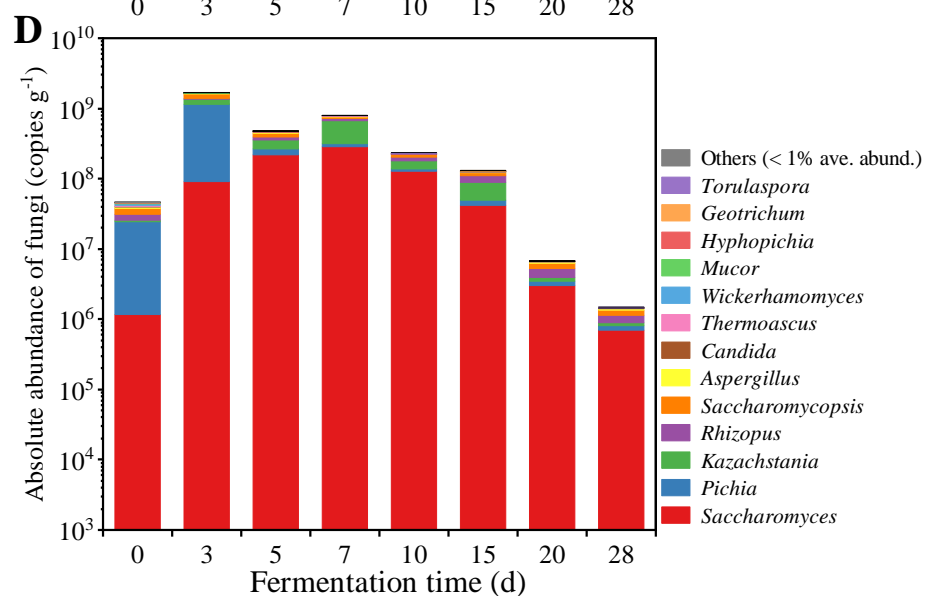

Supplement: FIG S6 [file mSystems.00964-20_sf006.pdf]
