# Supplementary material for: Gradient Internal Standard Method for Absolute Quantification of Microbial Amplicon Sequencing Data
Source: mSystems. 2021 Jan 12;6(1):e00964-20. doi: 10.1128/mSystems.00964-20 (PMC7901480; doi:10.1128/mSystems.00964-20)
Supplement: FIG S7 [file mSystems.00964-20_sf007.pdf]

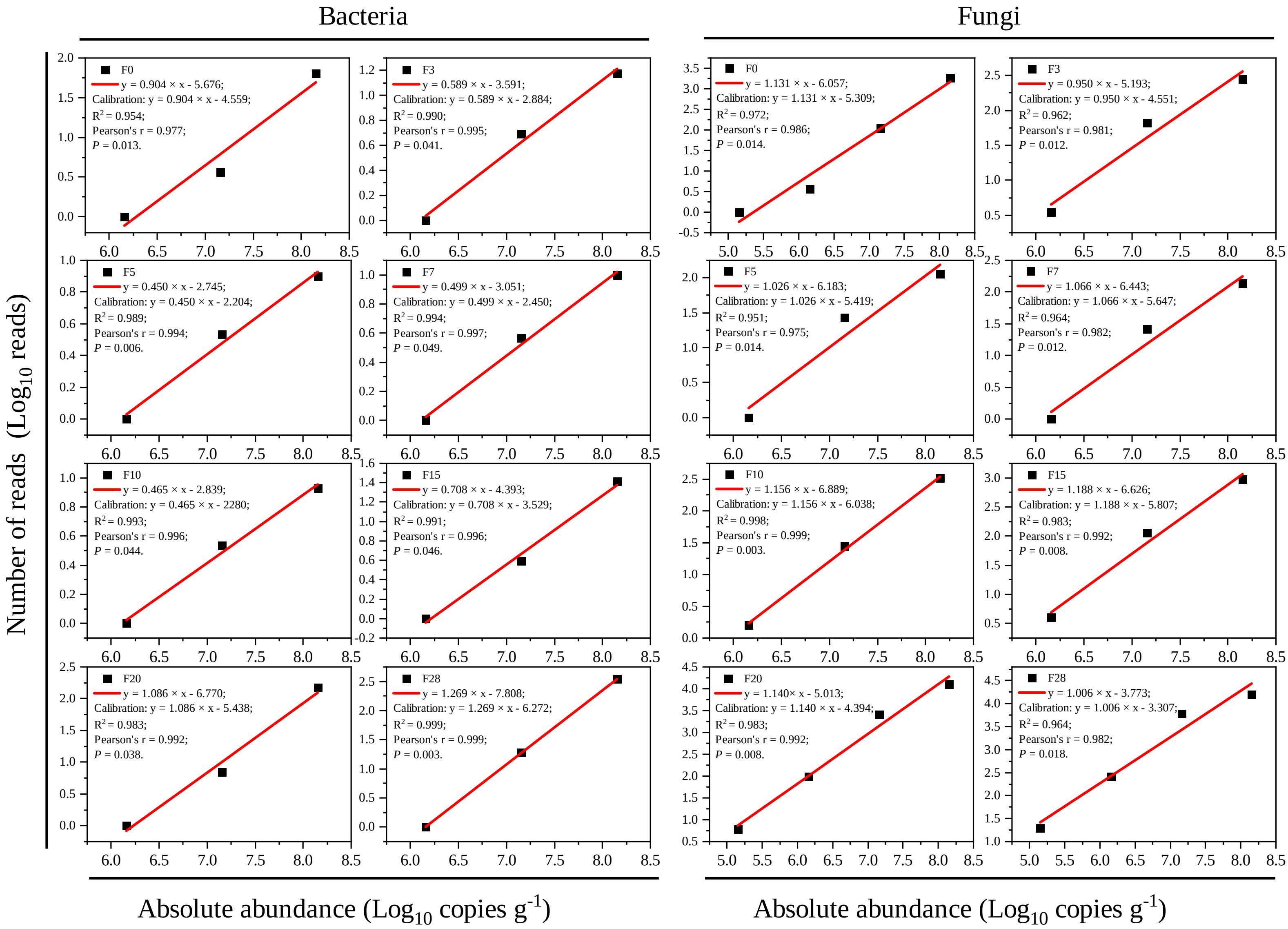

Bacteria
Fungi
Number of reads (Log10 reads)
F0
y = 0.904 × x - 5.676;
Calibration: y = 0.904 × x - 4.559;
R2 = 0.954;
Pearson's r = 0.977;
P = 0.013.
F3
y = 0.589 × x - 3.591;
Calibration: y = 0.589 × x - 2.884;
R2 = 0.990;
Pearson's r = 0.995;
P = 0.041.
F5
y = 0.450 × x - 2.745;
Calibration: y = 0.450 × x - 2.204;
R2 = 0.989;
Pearson's r = 0.994;
P = 0.006.
F7
y = 0.499 × x - 3.051;
Calibration: y = 0.499 × x - 2.450;
R2 = 0.994;
Pearson's r = 0.997;
P = 0.049.
F10
y = 0.465 × x - 2.839;
Calibration: y = 0.465 × x - 2280;
R2 = 0.993;
Pearson's r = 0.996;
P = 0.044.
F15
y = 0.708 × x - 4.393;
Calibration: y = 0.708 × x - 3.529;
R2 = 0.991;
Pearson's r = 0.996;
P = 0.046.
F20
y = 1.086 × x - 6.770;
Calibration: y = 1.086 × x - 5.438;
R2 = 0.983;
Pearson's r = 0.992;
P = 0.038.
F28
y = 1.269 × x - 7.808;
Calibration: y = 1.269 × x - 6.272;
R2 = 0.999;
Pearson's r = 0.999;
P = 0.003.
F0
y = 1.131 × x - 6.057;
Calibration: y = 1.131 × x - 5.309;
R2 = 0.972;
Pearson's r = 0.986;
P = 0.014.
F3
y = 0.950 × x - 5.193;
Calibration: y = 0.950 × x - 4.551;
R2 = 0.962;
Pearson's r = 0.981;
P = 0.012.
F5
y = 1.026 × x - 6.183;
Calibration: y = 1.026 × x - 5.419;
R2 = 0.951;
Pearson's r = 0.975;
P = 0.014.
F7
y = 1.066 × x - 6.443;
Calibration: y = 1.066 × x - 5.647;
R2 = 0.964;
Pearson's r = 0.982;
P = 0.012.
F10
y = 1.156 × x - 6.889;
Calibration: y = 1.156 × x - 6.038;
R2 = 0.998;
Pearson's r = 0.999;
P = 0.003.
F15
y = 1.188 × x - 6.626;
Calibration: y = 1.188 × x - 5.807;
R2 = 0.983;
Pearson's r = 0.992;
P = 0.008.
F20
y = 1.140× x - 5.013;
Calibration: y = 1.140 × x - 4.394;
R2 = 0.983;
Pearson's r = 0.992;
P = 0.008.
F28
y = 1.006 × x - 3.773;
Calibration: y = 1.006 × x - 3.307;
R2 = 0.964;
Pearson's r = 0.982;
P = 0.018.
Absolute abundance (Log10 copies g-1)
Absolute abundance (Log10 copies g-1)
